# Effects of Emotional Labor Factors and Working Environment on the Risk of Depression in Pink-Collar Workers

**DOI:** 10.3390/ijerph17145208

**Published:** 2020-07-19

**Authors:** Hae-ryoung Chun, Inhyung Cho, Youngeun Choi, Sung-il Cho

**Affiliations:** The Department of Public Health Science, Graduate School of Public Health, Seoul National University, Seoul 08826, Korea; mamimihae@gmail.com (H.-r.C.); ihcho04@snu.ac.kr (I.C.); yechoi92@gmail.com (Y.C.)

**Keywords:** emotional demand, service work, sales work, emotional display rules, health and safety information, The Korean Working Conditions Survey

## Abstract

Analyzing men and women separately, we examined the associations between six key elements of the psychosocial work environment of pink-collar workers (*n* = 7633) and the risk of depression, using logistic regression analysis with data from the Fifth Korean Working Conditions Survey (KWCS) conducted in 2017. We assessed the risk of depression according to the presence of emotional display rules (EDR), health and safety information (HSI), and emotional labor. In males, the risk of depression increased when there were no EDR and they had to interact with angry customers (OR 1.94, 95% CI 1.14–3.30). For women, the risk of depression increased if they had to interact with angry customers and EDR were present (OR, 1.73; 95% CI, 1.00–3.00), and if they did not receive HSI but had to interact with angry customers (OR, 1.66; 95% CI, 1.02–2.71), or hid their emotions and did not receive HSI (OR, 1.90; 95% CI, 1.50–2.40). The risk of depression increased more in the presence of EDR among women who hid their emotions (OR 1.80, 95% CI, 1.40–2.31) compared to women who did not hide their emotions and in the absence of EDR. Therefore, it is necessary to consider the effects of gender-specific factors on the risk of depression and revise current guidelines accordingly.

## 1. Introduction

Although major depressive disorder is prevalent worldwide, research investigating the risk of depression in the workplace is still insufficient [1]. A World Health Organization (WHO) study of the global burden of disease reported that management of depressive disorder is one of the most important goals of the 21st century [2]. Risk of depression had the third highest disease burden in 2004, and it is expected to have the highest burden by 2030 [2]. Risk of depression has a high prevalence and major impact, and the socioeconomic costs increase rapidly every year [3]. Risk of depression adversely affects job performance [4]. For example, risk of depression in the workplace can affect productivity by increasing absenteeism [5]. Sick leave due to mental health problems such as risk of depression has been increasing in recent years [6]. Also, when the mental health of those performing emotional work deteriorates, company productivity decreases, and excessive stress, risk of depression, anxiety, and suicidal thoughts can occur at the individual worker level [7,8]. As the workers’ health is important in the labor market, ensuring workers’ health and safety and attending to their working environment are crucial.

It is necessary to examine the working environment factors that affect the risk of depression among those pink-collar jobs associated with emotional labor, which have recently attracted increased interest [9]. Emotional labor is defined as the effort, planning, and control necessary to express the emotions required by an organization [10]. The term ‘pink collar’ refers to retail and service industry work [11]. As the size of the service sector grows, interest in service-related health issues and emotional labor is increasing [9]. In 2017, the rate of occupational injuries in the service industry increased by 12.5% compared to 2008. Interpersonal service workers are required to perform emotional labor and engage in superficial acts, making them subject to more work stress than non-service workers [12]. The term pink collar first appeared after World War II and was originally used to describe female workers in service jobs [13]. However, in modern times, the definition has been expanded to include service jobs and sales occupations not specific to women [14]. There is no standardized definition of a pink-collar occupation [13]. Interest in pink-collar occupations has increased, as the industrial sector has recently changed its focus from manufacturing to service [15]. Furthermore, many large and small companies are allocating more workers to customer service departments [16].

Emotional labor has become an important concept when discussing working conditions in Korean society as the service industry expands and the number of workers involved in emotional labor increases accordingly [7]. In addition, human rights violations and acts of workplace bullying, such as mistreatment by aggressive customers and power abuse by superiors, which are inherent in Korean society, have become issues, social empathy and interest have increased. As emotional workers’ mental health problems have emerged and interest in them has increased, several guidelines have been implemented. On 7 January 2016, the Seoul City Ordinance was enacted to protect emotional workers’ rights. A Comprehensive Plan for Emotional Labor were established on 1 January 2016, the guide for emotional workers was published in June 2016, and an emotional labor center was established in October 2016. When such policies are implemented, they apply to all occupational groups, including those performing emotional labor. Therefore, it is important to identify factors affecting the risk of depression in pink-collar occupational groups across all service and sales positions rather than simply considering specific occupational groups, as in previous studies.

Although risk of depression has many causes, this study aimed to investigate the association of six key elements of the psychosocial work environment with the risk of depression. According to a previous study described below, several factors affect the risk of depression in the workplace: lower age and a lack of social support at work [17]; high job demands [18]; long working hours, part-time work, smoking habits, lack of sleep, lack of exercise, salty eating habits, alcohol consumption, and dealing with aggressive customers [19,20]. According to the European Workplace Safety and Health Administration (EU-OSHA), six key factors associated with the economic and social work environment cause serious mental health problems. Direction de l’Animation de la Recherche, des Etudes et des Statistiques (DARES, the research arm of the French Ministry of Work and Employment) identified six key elements of the psychosocial work environment: high work demands, emotional demands, lack of autonomy, ethical conflicts, low-level social relations, and job insecurity [21].

When dealing with customers [16], pink-collar workers conduct emotional labor according to the emotional display rules (EDR) conveyed by their company [22]. Previous empirical studies demonstrated that emotional labor has both positive and negative effects on health [23]. The exact manner in which emotional labor negatively affects mental health has not been fully investigated [16]. Emotional labor facilitates work efficiency and self-expression and can have a positive effect on workers [24]. However, due to emotional control by the company providing the emotional work [25], workers may experience emotional dissonance if they have to hide their emotions. Emotional dissonance entails discrepancies between the emotion felt by the person and that displayed [10,12]. Emotional dissonance can lower well-being and lead to health complaints [19], burnout [23], job stress, risk of depression [16,26], self-alienation [24], suicidal thoughts [27], and fatigue [28]. Therefore, job exit and increased job dissatisfaction depend not only on the fact that one is engaging in emotional labor or on the amount and frequency of emotional work performed but also on the effects of such dissonance [28].

Provision of health and safety information (HSI) by employers, which can protect pink-collar workers from injury, disease, and morbidity, is crucial, but is often not actively implemented. Article 41 of the revised Occupational Safety and Health Act, called the Emotional Workers Protection Act, came into effect on 18 October 2018. Furthermore, in accordance with Article 669 of the Occupational Safety and Health Act, measures must be taken to prevent health disorders caused by occupational stress when dealing with high physical and mental stress. However, a survey on emotional labor and harassment in the workplace in 2019, which was conducted a year after the law was implemented, found that 70% of 2765 emotional workers said they were unprotected. Most research and intervention regarding HSI focuses on manufacturing or secondary industries [1] and rarely targets pink-collar workers [29]. There is also a lack of research examining the link between HSI and mental health [29]. Therefore, it is necessary to assess whether employers protect emotional workers by providing HSI.

In Korean society, differences in the social status and stereotypes of men and women remain; therefore, elements of the working environment that affect the risk of depression should be assessed separately according to gender. Women are much more likely to perform emotional labor than men, and the demand for their work is higher [30]. There is a deep-routed stereotype that women are more suited to particular jobs than men [31]. By 2010, women’s median earnings were more than 80% of men’s in most countries, but in Korea, women’s earnings were only 63% of men’s [32]. The rate of female labor force participation is only 59%, compared with 79% for men [33]. The boards of directors of Korean listed companies are 98% male; only one of 109 companies has a female chief officer [33]. Various studies have shown that low job empowerment is associated with emotional fatigue [34] and mental illness [35]. Therefore, the impact of EDR on female workers is greater than that on male workers because it places a greater emotional burden on women due to their low level of job control [16].

The present study used data from the Fifth Korean Working Conditions Survey (KWCS) conducted in 2017 to examine the association between the six key factors of the psychosocial work environment identified by DARES and the occurrence of risk of depression among pink-collar workers. We analyzed risk of depression using the WHO-5, which is a structured questionnaire that provides a well-being index. The research hypotheses investigated in this study were as follows. First, the six key factors of the psychosocial work environment are associated with risk of depression in pink-collar workers. Second, the associations between these six key factors and risk of depression vary with gender. The risk of depression varies depending on emotional labor factors (hiding emotions and dealing with angry customers) and the provision of guidelines on EDR and HSI). Factors that affect the risk of depression should be identified so that the work environment can be improved in the future. The EDR and HSI may also need to be revised in some cases.

## 2. Materials and Methods

### 2.1. Data Sources and Participants

**Figure 1:**
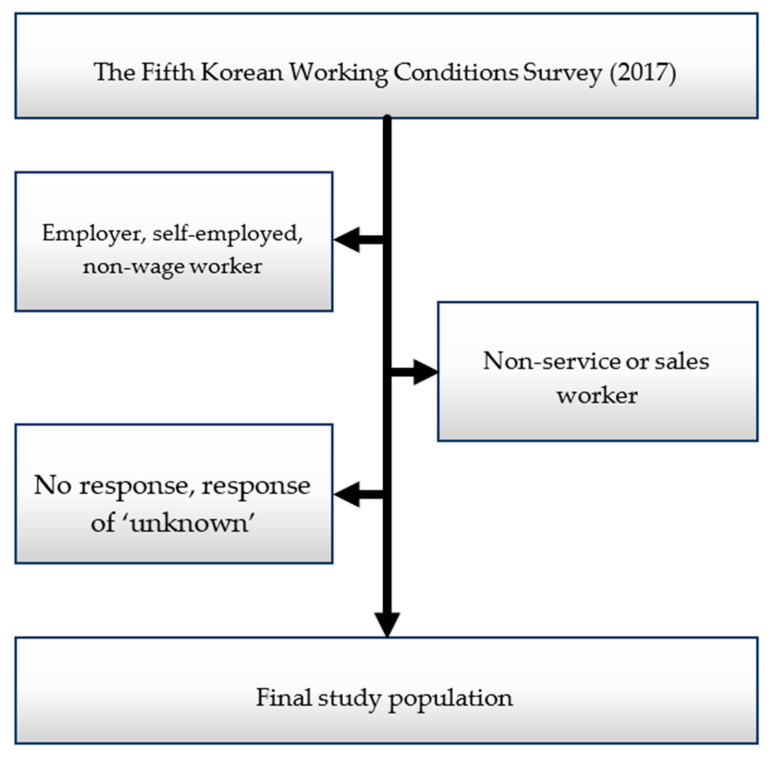
Schematic of the study participant selection process.

This study used data from the Fifth KWCS, collected from respondents aged ≥15 years. The KWCS was developed based on the original questionnaire of the European Working Conditions Survey (EWCS), which was conducted in Europe in 2015; it is now available in two languages: Korean and English. The KWCS data are secondary data available after receiving permission from the Korea Occupational Safety & Health Agency. The data can be downloaded from the Occupational Safety and Health Research Institute website (https://oshri.kosha.or.kr/). This ongoing survey, which began in 2006, was approved by the Korean government. The validity and reliability of the KWCS data were confirmed in a previous study [36]. This study is an interview survey based on the probability proportional to size systematic sampling. The questionnaire is administered by an interviewer, who asks the questions and records the responses directly. For the Fifth KWCS, the response rate was 0.449, the cooperation rate was 0.640, and the refusal rate was 0.253 [37]. The survey gathered data from 50,205 workers, excluding 80,440 who declined to respond or gave incomplete answers [38]. As shown in Figure 1, subjects were selected for the current study according to the following inclusion criteria to provide the most suitable sample for the research: (1) paid workers; (2) answered all relevant survey questions; and (3) engage in customer service or sales operations. Self-employed respondents, employers, and non-wage workers were excluded (*n* = 20,097). Among the wage workers, those who were not in service or sales positions (*n* = 21,023) and those who did not answer all of the questions or responded with ‘unknown’ (*n* = 1452) to some items were also excluded. After this selection process, 7633 subjects (2460 men and 5173 women) were included in the analysis. The study was approved by the Institutional Review Board (IRB) of Seoul National University (IRB No. E1909/003-006).

### 2.2. Measures

#### 2.2.1. Pink-Collar Occupations

Most published studies have been conducted only with service workers or sales workers engaged in emotional labor or in specific occupations, and research on pink-collar workers performing emotional service and sales jobs is lacking. Therefore, this study selected pink-collar workers, the occupational group encompassing service workers and sales workers in the Korea Standard Classification of Occupations. In response to the question, “Which of the following best describes your job?” those who responded service worker or sales worker were selected.

#### 2.2.2. Six Key Factors of the Psychosocial Work Environment

DARES previously classified six key factors that affect the psychosocial work environment [21]; KWCS included questions related to these six key factors. According to the DARES report [39], the six key factors are high work demands, emotional demands, lack of autonomy, ethical conflicts, low-level social relations, and job insecurity.

High work demands are considered to exist when there are EDR, and emotional labor is determined by the requirements to hide emotions and interact with angry customers. Lack of autonomy is defined herein as the lack of opportunity to apply one’s own ideas at work. Ethical conflict arises when the work being performed is not perceived as useful. Low-level social relations refers to a lack of support from one’s boss or colleagues, and is also affected by the availability of HSI and perception of treatment by the company in the case of conflict. Job insecurity depends on whether the employee believes that a new job could be obtained easily after leaving the company.

##### High Work Demands

Work demands are defined as the cognitive and physical effort that a person must make to perform his or her work in terms of quantity, speed, and personality traits [21]. Job demands are related to health impairment [18]. High work demands, which occur when the labor demands are too great [40], can adversely affect health [41].

In this study, high work demands depended on the presence of EDR. EDR comprises regulations governing behavior that the company requires of emotional workers where social interactions with customers, clients, or patients are a significant part of the job [12]. Whether participants had been told about EDR was determined by the question “Does the company have any emotional rules regarding your work?” Participants selected “Yes” or “No.” Subjects who answered “yes” were those who received communications about EDR during working hours, and those who answered “no” did not. Participants who answered “Unknown” and those who failed to answer were removed.

##### Emotional Demands

With emotional and psychological work, workers are expected to respond to customers in an emotionally appropriate way according to the requirements of the company [30]. When dealing with customers, workers need to hide their personal feelings, such as suppressing fear or maintaining kindness. Excessive emotional demands negatively affect mental health [42] and increase the risk of suicidal thoughts [27].

**Engaging with Angry Customers:** Both men and women deal with angry customers, and the risk of depression increases when EDR is applied [16]. Here, the study subjects were asked, “Does your work involve dealing with angry customers or patients?” Individuals who responded “always” and “almost always” were regarded as dealing with angry customers. Conversely, those who indicated that they worked with angry customers “3/4 of my working hours,” “half of my working hours,” “1/4 of my working hours,” “almost never,” or “never” were classified as not dealing with angry customers.

**Hiding Emotions:** When performing emotional work, the worker is controlled by the EDR suggested by the organization; if there is a discrepancy between the worker’s actual emotion and the requested emotional state, emotional dissonance can occur [30]. Hiding or suppressing emotions can cause psychological stress [43]. Indeed, hiding emotions is directly related to increased job stress and indirectly associated with risk of depression [26]. Possible answers to the question “I must hide my emotions at work” were as follows: “Always”, “Mostly”, “Sometimes”, “Not often”, “Not at all”, “Not applicable” and “Unknown/No response”. Responses were divided into two categories based on the degree to which emotions were hidden: hiding emotions (always yes, mostly yes) and not hiding emotions (not often, not at all, not applicable).

##### Lack of Autonomy

Lack of autonomy may occur if people do not have the opportunity to fully demonstrate their capabilities. High demands and low decision-making autonomy have a strong impact on the risk of depression [44] and are also linked to mental health problems [45]. We assessed autonomy by asking participants to respond to the statement, “I can apply my thoughts to my work.” The available answers were “Always”, “Most of the time”, “Sometimes”, “Not often”, “Not at all”, “Not applicable”, and “Unknown/No response” We reclassified workers into two groups: those who felt able to apply their thinking to their work (always, most of the time) and those who did not (sometimes, not often, not at all).

##### Ethical Conflicts

Conflicts of values refer to ethical conflicts and unnecessary work. When an individual feels that their work is useless, it creates personal stress and can also lead to health problems [46]. All participants responded to the statement, “I think I am doing something useful” as a measure of ethical conflict. The available answers were “Always”, “Usually”, “Sometimes”, “Not often”, “Not at all”, “Not applicable”, and “Unknown/No response”. Participants were classified as feeling as if their work was useful (always, usually) or not (sometimes, not often, not at all).

##### Low-Level Social Relations

Social relations at work involve coworkers, bosses, relationships outside the company, and violence within the company. Dealing with work stress and related burdens can be difficult without support from the boss and colleagues and a sense of belonging within the company [47,48]. A negative relationship between the boss and subordinates significantly affects the likelihood of depression [49]. To assess low-level social relations, we investigated whether workers were supported by their boss and colleagues, and whether HSI was provided. Fair conduct of the company when conflicts arose was also checked for.

**Receiving Support from the Boss:** We asked all participants to respond to the following statement about their work situation: “My boss helps and supports me.” The available answers were “Always”, “Usually”, “Sometimes”, “Not often”, “Not at all”, “Not applicable”, and “Unknown/No response”. Participants were divided into two groups: those receiving support from their boss (always, usually) and those not receiving such support (sometimes, not often, not at all).

**Receiving Support from Colleagues:** All participants were also asked to respond to the statement “My colleagues help and support me” using the following options: “Always”, “Usually”, “Sometimes”, “Not often”, “Not at all”, “Not applicable” and “Unknown/No response”. The respondents were then divided into those receiving support (always, usually) and those not receiving support (sometimes, usually not, not at all).

**Health and Safety Information (HSI):** In accordance with Article 31 of the Occupational Safety and Health Act, employers should regularly provide information related to safety and health, including information on accident prevention, health management, disaster cases, and preventive measures. Previous studies demonstrated that there is a high probability of injury in the workplace when HSI is not provided, but few reports have examined the effects of such provision on mental health [29]. In response to the question, “Did you receive information regarding the health and safety risks associated with your job performance,” subjects were divided into those who were “Well-informed” and “Not informed.” If the job was associated with a risk of injury, the participant was asked whether information about the likelihood of mental and physical injury had been provided.

**Handling Conflicts in A Fair Way:** Here, the answers to the question “Is conflict handled in a fair way?” were “Always”, “Usually”, “Sometimes”, “Not often”, “Not at all”, “Not applicable” and “Unknown/No response”. Participants were sorted into two categories: those who thought they were being treated fairly (always, usually) and those who thought they were being treated unfairly (not often, not at all). Subjects who identified the question as not applicable or the answer as unknown/no response were removed from subsequent analysis.

##### Job Insecurity

Job insecurity refers to anxiety about the employment situation, whether related to employment status, wages, career security, or job sustainability. The fear of losing the job and a lack of career prospects can have a major impact on workers’ well-being [50,51], and job insecurity is highly related to the risk of depression [52]. The following statement about job insecurity was presented: “Even if I quit or lose my job, I can easily find a job that gives me a similar wage.” The available answers were “Highly agree”, “Generally agree”, “Somewhat agree”, “Generally disagree”, “Completely disagree”, “Not applicable”, “Do not know” and “No response”. The subjects were categorized as those who felt that they could get a new job (highly agree, generally agree, somewhat agree) and those who did not (generally disagree, completely disagree).

#### 2.2.3. Risk of Depression

Previous studies have mainly used self-reported mental health questionnaires to examine factors affecting the mental health of emotional workers. Therefore, the judgment may be inaccurate. However, the current study used the WHO-5 Well-Being Index, which is a structured questionnaire that has sufficient internal and external validity to screen for risk of depression [53,54]. The index was first introduced in its current form in 1998 as part of the DepCare project on welfare measures in primary healthcare at a WHO regional office in Europe. It evaluates respondents’ feelings for 2 weeks and consists of the following five statements: (1) I have felt cheerful and in good spirits; (2) I have felt calm and relaxed; (3) I have felt active and vigorous; (4) I woke up feeling fresh and rested; and (5) My daily life has been filled with things that interest me. The responses for each item are scored according to the following scale: (5) All of the time; (4) Most of the time; (3) More than half of the time; (2) Less than half of the time; (1) Some of the time; and (0) At no time. The scores for all questions are added, and a total score of 13 or less indicates poor mental health and suggests the subject should be tested for risk of depression.

#### 2.2.4. Key Covariates

The following sociodemographic characteristics were evaluated: age (15–29, 30–39, 40-49, 50–59, ≥60 years), education (middle school or less, high school graduation, college graduation), and area of residence (big city or small city). Job status was divided into regular and temporary jobs. The average number of hours per week spent working in customer relations was 43 h, and the average number of working hours for 5-day work weeks was 49 h and 55 min. Therefore, we classified working hours as follows: <43 h, 43–50 h, and ≥50 h. In 2017, the average monthly salary of wage workers was 2.87 million won (US$ 2337), and the median salary was 2.1 million won (US$ 1710). Most study participants received wages of 1.5–2.5 million won, followed by <0.85 million won and 0.85–1.5 million won. Therefore, we classified salary as <1.5 million won, 1.5–2.5 million won, 2.5–4.0 million won, and ≥4.0 million won).

### 2.3. Statistical Analysis

The data of male and female workers were analyzed separately. The demographic characteristics related to pink-collar workers, general working conditions, and the six key factors were analyzed using descriptive statistics. Data on the number and proportion (%) of workers (*n*) are provided. Standardized weights were applied to the proportions. Chi-square tests were conducted to confirm the risk of depression according to the pink-collar working characteristics, basic working conditions, and six key factors delineated above. Logistic regression was performed to examine the effects of six key factors on the risk of depression among pink-collar workers. Odds ratios and 95% confidence intervals were calculated for the six key factors (high work demands, emotional demands, lack of autonomy, ethical conflicts, low-level social relations, and job insecurity). The analyses were adjusted for general characteristics (age, education level, city), basic working conditions (working hours per week, monthly salary, working status), and nine other variables. Interaction analyses were conducted to determine the effects on the risk of depression of EDR, provision of HSI, requirement to hide emotions, and the expectation that one should deal with angry customers. All analyses were performed using the SURVEYFREQ and SURVEYLOGISTIC procedures of SAS software (ver. 9.4) (SAS Institute Inc., Seoul, South Korea), with the survey weights provided.

## 3. Results

### 3.1. Demographics, Working Conditions, and the Risk of Depression Among Pink-Collar Workers

Table 1 shows the general characteristics and working conditions of pink-collar workers and the risk of depression. The highest proportion of male workers were in their 30 s (29.84%), whereas women in their fifties had the largest proportion (26.03%). However, the highest risk of depression was observed in subjects aged 60 years and older for both men (33.87%, *p* < 0.01) and women (30.12%, *p* < 0.01). With regard to education, most males were college graduates (61.86%), whereas the highest proportion of females were high school graduates (51.21%). However, risk of depression was most common among males (28.85%, *p* = 0.02) and females (33.12%, *p* < 0.01) with only middle school education. Larger proportions of men (40.63%) and women (55.96%) worked less than 43 h than any other work hours category, and men (30.17%, *p* < 0.01) who worked for more than 50 h had the highest risk of depression.

The most common wage among males (40.64%) was 2.5–4 million won, and that among women was 1.5–2.5 million won (48.49%). Most men (73.78%) and women (73.49%) reported that they were not subject to EDR. Most men (95.07%) and women (94.02%) reported that they did not have to interact with angry customers, but the risk of depression was higher only in males (33.00%, *p* = 0.02) who had to interact with such customers. Among males, 52.88% of respondents said that they could apply their thoughts to their work, whereas 51.26% of women responded that they could not do so. However, the risk of depression was high among both men (30.73%, *p* < 0.01) and women (31.97%, *p* < 0.01) who could not apply their own ideas at work. A high proportion of men (54.16%) and women (52.15%) felt that they were doing useful work, but men (32.92%, *p* < 0.01) and women (32.43%, *p* < 0.01) had a significantly higher risk of depression when they thought they were not doing useful work.

Most men (62.18%) and women (61.46%) reported that their company provided HSI. The risk of depression was highest among the men (29.48%, *p* < 0.01) and women (30.75%, *p* < 0.01) who reported that their companies provided no HSI. High proportions of both men (56.50%) and women (54.83%) answered that their company handled conflict in a fair manner. However, the risk of depression was higher among respondents who said that they were not being treated in a fair manner (women, 33.37%, *p* < 0.01; men, 32.28%, *p* < 0.01). Most men (66.06%) and women (72.38%) stated that they could easily find a new job even if they quit their current work, only women (27.35%, *p* = 0.01) were found to have increased risk of depression when they were unable to find a new job after quitting work.

### 3.2. Association between the Six Key Factors of the Psychosocial Work Environment and the Risk of Depression in Both Genders

As shown in Table 2, after adjusting for demographic variables (such as age, residential area, education level), basic working conditions (such as employment status, working hours and wages), and the other key factor sub-variables, the association of each key factor sub-variable with risk of depression was examined using logistic regression analysis. Table 2 shows association between the risk of depression and six key factors through Odds Ratio (OR) and 95% Confidence Interval(CI), whereas Table S1 shows the beta coefficient and p value as well as OR and 95% CI. Unlike in women, the risk of depression in men was higher when they were expected to work with angry customers (OR 1.66, 95% CI 1.05–2.64).

Unlike men, women had an increased risk of depression when they knew of EDR (OR 1.26, 95% CI 1.03–1.53), when they had to hide their emotions (OR 1.36, 95% CI 1.15–1.62), when they experienced no support from their colleagues (OR 1.58, 95% CI 1.30–1.93), and if HSI was not given (OR 1.40, 95% CI 1.18–1.66).

The risk of depression increased significantly among males and females who did not perceive their work as useful (OR 1.67, 95% CI 1.27–2.21 and OR 1.56, 95% CI 1.29–1.87, respectively) and thought that conflict was not handled fairly by the company (OR 1.75, 95% CI 1.35–2.28 and OR 1.44, 95% CI 1.20–1.72, respectively). Only the effect of key factor sub-variables, including hiding emotions (*p* = 0.04) and support from colleagues (*p* = 0.03), on the risk of depression differed by gender.

### 3.3. Interactions among EDR, HSI, and Emotional Demands

As shown in Table 3, we examined how the six key factors affect the risk of depression depending on whether a job is emotionally demanding and whether the company has EDR and/or provides HSI. In males not subjected to EDR, the risk of depression increased if they interacted with angry customers, compared with those who did not need to interact with angry customers (OR 1.94, 95% CI 1.14–3.30). In men, there was no significant association between EDR and risk of depression, although there was an interaction effect between EDR and dealing with angry customers.

Compared with women who do not experience EDR and did not deal with angry customers, the risk of depression increased most when their work included both EDR and angry customers (OR 1.73, 95% CI 1.00–3.00). The risk of depression also increased among women who do not deal with angry customers and were subjected to EDR (OR 1.24, 95% CI 1.01–1.52). Women who needed to hide their emotions (OR 1.24, 95% CI 1.01–1.51) had a higher risk of depression than those who were not subjected to EDR and did not hide their emotions. The risk of depression was higher in women subjected to EDR and hiding their emotions (OR 1.80, 95% CI 1.40–2.31) than in those who did not hide their emotions and were not subject to EDR.

In women who were provided HSI, hiding emotions increased their risk of depression (OR 1.46, 95% CI 1.16–1.83). In addition, the risk of depression increased further when HSI was not provided compared with those who were given HSI and did not hide emotions (OR 1.51, 95% CI 1.19–1.92). The risk of depression was greatest when HSI was not provided and emotions needed to be hidden (OR 1.90, 95% CI 1.50–2.40). Among women, the risk of depression increased if they were not given HSI, regardless of whether they were dealing with angry customers compared with the situation when they were given HSI and did not deal with angry customers. Participants who did not deal with angry customers and did not receive HSI also had a higher risk of depression (OR 1.41, 95% CI 1.18–1.68). Finally, the risk of depression was high among those who dealt with angry customers without being provided HSI (OR 1.66, 95% CI 1.02–2.71).

## 4. Discussion

The first hypothesis of this study was that the six key factors in the psychosocial work environment are associated with the risk of depression among service and sales workers. The second hypothesis was that the associations between these six key factors and the risk of depression vary by gender. The results showed that some of the six key factors were significantly associated with the risk of depression, and some of the associations differed by gender. The third hypothesis was that emotional labor factors and emotional labor-related guidelines affect the risk of depression. The results revealed an interaction effect between emotional labor factors (hiding emotions, dealing with angry customers) and guidelines pertaining to EDR/HSI on the risk of depression, which varied by gender. These findings are explained in more detail below.

The risk of depression according to demographic characteristics, distribution of working conditions, and the six key factors differed by gender. Women in the Korean working environment have a lower social status and less job control than men, and there is also an extreme wage gap between men and women in Korea. Unlike males, many female respondents said that they had to hide their feelings and could not apply their thoughts to their work. Both male and female respondents who were not told of EDR showed a high frequency of risk of depression. Unlike men, women who knew of EDR had a higher risk of depression.

After adjusting demographic variables (age, residence, education level) and basic working conditions (employment status, working hours, wages), the effects of the six main factors on the risk of depression for men and women differ. Among men, dealing with angry customers was significantly associated with an increased risk of depression. Women who were subjected to EDR, had to hide their feelings, could not work autonomously, did not receive support from their colleagues, and did not receive HSI had a higher risk of depression. The risk of depression increased in both sexes when they thought they were not being treated fairly when a conflict arose or were engaged in work that they did not perceive as useful.

The interaction analysis examined differences in the risk of depression according to emotional labor factors and whether guidelines were provided. In men, the risk of depression increased when dealing with angry customers and knowing of EDR. No other interactions were significant. In women, all interactions tended to be similar to those reported in previous studies. Dealing with angry customers, being aware of EDR, hiding emotions, and not receiving HSI had additive effects on the risk of depression.

Employers should be aware of the factors increasing the risk of depression in men and women. In addition, the working environment should be improved, and guidance should be provided to protect workers. The current study revealed that when considering the factors that affect the risk of depression in men and women, it is important that EDR and HSI be managed differently according to gender. This is consistent with previous studies demonstrating that women in the Korean working environment have a lower social status than men [32,33]. When dealing with angry customers, the company should provide step-by-step measures to both men and women, without unconditionally compelling the worker to suppress feelings or apologize to the customer. Also, consistent with the revised occupational safety and health law, workers should not be treated unfairly when conflict arises. In particular, the company should not force emotional regulation on women who are sensitive about having to hide their emotions. In addition, HSI should be communicated more actively than is currently the case. In particular, women place more importance on relationships with colleagues than men, which can influence life satisfaction [55]. Therefore, systems and working conditions should also be improved to provide mental health care support and a supportive environment among colleagues [56]. By revising EDR and providing HSI, many companies can further protect workers’ safety and health [57].

There are some limitations to this study. First, it was based on cross-sectional data; therefore, the causal relationship between the six key factors and the risk of depression could not be examined. However, previous longitudinal studies demonstrated that adverse psychosocial working conditions, such as the variables assessed in the current study, increase the risk of depression [58]. Second, this study treated the demographic and basic working condition factors only as potential confounders, such that they were adjusted for in the analysis of the effects of the six key factors on risk of depression. It may be worthwhile to determine whether these factors also act as intermediate pathways to the risk of depression in future studies. Third, the factors used to characterize the working environment were subjective. Therefore, some limitations such as recall bias may be present. The variables of interest were those questioning the worker’s perception of whether a working condition was important. Hence, the structured WHO-5 Well-Being Index was used. Fourth, because this study used data from 2017, it is not possible to know the effect on the risk of depression of the amendment to the 2018 Occupational Safety and Health Act. However, even before 2018, there were laws for emotional workers, and EDR and HSI were required. This means that looking at the effects of policy efforts on emotional workers before the revision of the law still allows for the examination of pink-collar workers. Despite these limitations, one of the strengths of this study is that we investigated the associations between the six key factors and the risk of depression in a nationally representative Korean sample. The data are suitable for analysis because KWCS included a variety of factors affecting industrial accidents. Also, because KWCS is conducted every three years, the 6th KWCS could be used to examine the mental health effects of the revised Occupational Safety and Health Act on pink collar workers.

## 5. Conclusions

In this study, the effects of the six key elements of the psychosocial work environment on risk of depression in pink-collar workers in Korea were examined according to gender using the KWCS. For men, the risk of depression increased when dealing with angry customers. In women, the risk of depression increased when they were subjected to EDR, needed to hide their emotions, could not apply their ideas to their work, or did not think perceive their work as useful. The risk of depression also increased when no HSI or support from colleagues was provided, and when they were not dealt with fairly in conflict situations by the company. In men, the risk of depression was highest when EDR was provided and the worker had to deal with angry customers; no other interaction analysis showed significant results. For women, the risk of depression increased when they had to hide their emotions, dealt with angry customers, knew of EDR, and did not receive HSI. To reduce the risk of depression, HSI and EDR should be revised in ways that protect workers and should be distributed more widely. This study should be used as a basis to improve the occupational environment and its effects on the mental health of pink-collar workers.

## Figures and Tables

**Table 1:** The numbers and percentages with the risk of depression in men and women according to general characteristics.

			Males (*n* = 2460)			Females (*n* = 5173)		
			Total	Depressed	*p* *	Total	Depressed	*p* *
			*n* (% ^1^)	*n* (% ^2^)		*n* (% ^1^)	*n* (% ^2^)	
Demographics								
Age (y)		15–29	678 (26.87)	129 (19.75)	<0.01	702 (23.60)	127 (17.12)	<0.01
		30–39	753 (29.84)	197 (27.91)		778 (16.40)	182 (23.84)	
		40–49	539 (22.53)	134 (26.31)		1332 (22.98)	354 (24.74)	
		50–59	342 (15.32)	83 (23.43)		1716 (26.03)	496 (30.10)	
		60+	148 (5.45)	44 (33.87)		645 (10.99)	211 (30.12)	
City size		Big	1256 (49.13)	271 (23.70)	0.1	2628 (49.2)	671 (25.45)	0.3
		Small	1204 (50.87)	316 (26.25)		2545 (50.8)	699 (24.12)	
Education level		Middle school or lower	80 (2.79)	22 (28.85)	0.02	445 (7.00)	165 (33.12)	<0.01
		High school	906 (35.35)	236 (27.65)		2847 (51.21)	774 (26.11)	
		College or higher	1474 (61.86)	329 (23.31)		1881 (41.79)	431 (21.75)	
Working conditions								
Work time (h)	<43		985 (40.63)	195 (21.07)	<0.01	2729 (55.96)	721 (24.94)	0.57
	43–50		507 (20.30)	121 (22.90)		1035 (18.14)	253 (23.41)	
	>50		968 (39.07)	271 (30.17)		1409 (25.90)	396 (25.39)	
Wages ^3^	<1.5		289 (9.91)	52 (17.32)	0.01	1419 (30.06)	381 (25.13)	0.34
	1.5–2.5		727 (28.44)	181 (25.18)		2625 (48.49)	700 (25.19)	
	2.5–4.0		968 (40.64)	240 (26.58)		729 (13.91)	170 (21.93)	
	>4.0		476 (21.00)	114 (25.31)		400 (7.54)	119 (26.00)	
Employment status	Regular		2028 (82.59)	479 (24.69)	0.4	3746 (69.75)	947 (23.40)	<0.01
	Temporary		432 (17.41)	108 (26.43)		1427 (30.25)	423 (27.96)	
Six key factors in the psychosocial work environment								
High work demands		No EDR	1843 (73.78)	442 (25.98)	0.04	3837 (73.49)	1003 (24.28)	0.19
		EDR	617 (26.22)	145 (22.23)		1336 (26.51)	367 (26.16)	
Emotional demands		No need to hide emotions	1326 (53.07)	328 (25.60)	0.41	2600 (50.58)	706 (24.01)	0.23
		Hide Emotions	1134 (46.93)	259 (24.31)		2573 (49.42)	664 (25.53)	
		No angry customers	2344 (95.07)	554 (24.58)	0.02	4855 (94.02)	1286 (24.57)	0.2
		Angry customers	116 (4.93)	33 (33.00)		318 (5.98)	84 (28.04)	
Autonomy		No	1181 (47.12)	355 (30.73)	<0.01	2656 (51.26)	923 (31.97)	<0.01
		Yes	1279 (52.88)	232 (19.89)		2517 (48.74)	447 (17.21)	
Ethical conflict		Not useful	1143 (45.84)	360 (32.92)	<0.01	2483 (47.85)	865 (32.43)	<0.01
		useful	1317 (54.16)	227 (18.29)		2690 (52.15)	505 (17.76)	
Social relations and company conduct		No boss support	935 (37.84)	297 (30.99)	<0.01	2108 (40.47)	743 (32.43)	<0.01
		Boss support	1525 (62.16)	290 (21.35)		3065 (59.53)	627 (19.57)	
		No colleague support	865 (34.00)	273 (31.59)	<0.01	1893 (36.13)	687 (34.82)	<0.01
		Colleague support	1595 (66.00)	314 (21.60)		3280 (63.87)	683 (19.10)	
		No HSI	942 (37.82)	260 (29.48)	<0.01	2024 (38.54)	674 (30.75)	<0.01
		HSI	1518 (62.18)	327 (22.27)		3149 (61.46)	696 (21.03)	
		Unfair	1081 (43.50)	348 (33.37)	<0.01	2393 (45.17)	832 (32.28)	<0.01
		Fair	1379 (56.50)	239 (18.55)		2780 (54.83)	538 (18.59)	
Job insecurity		No new job prospects	795 (33.94)	222 (26.22)	0.26	1485 (27.62)	451 (27.35)	0.01
		New job prospects	1665 (66.06)	365 (24.37)		3688 (72.38)	919 (23.79)	

* *p*-values from chi-square test. ^1^ The weighted percentages for each variable in the total column. ^2^ The weighted percentages for depression risk in the groups of variable. ^3^ Million Korean won (KRW); 1228 KRW = 1 US dollar (as of June 2020).

**Table 2:** Six key factors associated with risk of depression.

		Males (*n* = 2460)			Females (*n* = 5173)		
		OR *		95% CI *		OR *	95% CI *
Demographics							
Age (y)	30–39	1.66 ‡		1.13–2.44		1.66 ‡	1.19–2.31
	40–49	1.57 †		1.05–2.36		1.82 ‡	1.35–2.46
	50–59	1.33		0.85–2.07		2.33 ‡	1.73–3.14
	60+	2.13 ‡		1.16–3.90		2.32 ‡	1.58–3.42
	15–29	1.00				1.00	
City size	Small	1.19		0.94–1.51		0.96	0.81–1.13
	Big	1.00				1.00	
Education level	High school	1.08		0.51–2.28		1.28	0.88–1.86
	College or higher	1.2		0.90–1.60		0.98	0.80–1.20
	Middle school or lower	1.00				1.00	
		Working Conditions					
Work time (h)	43–50	1.03		0.72–1.46		0.93	0.74–1.16
	>50	1.33 †		1.00–1.78		1.02	0.83–1.25
	<43	1.00				1.00	
Wages ^1^	1.5–2.5	1.78 †		1.01–3.12		1.12	0.90–1.40
	2.5–4.0	2.1 ‡		1.18–3.72		1	0.73–1.35
	>4.0	2.19 ‡		1.19–4.05		1.26	0.88–1.81
	<1.5	1.00				1.00	
Employment status	Temporary	1.49		0.99–2.23		1.33 ‡	1.09–1.62
	Regular	1.00				1.00	
Six key factors	Variables						
High work demands	EDR		1.01	0.76–1.34		1.26 †	1.03–1.53
	No EDR		1.00			1.00	
Emotional demands	Hide emotions **		1.00	0.78–1.28		1.36 ‡	1.15–1.62
	No need to hide emotions		1.00			1.00	
	Angry customers		1.66 †	1.05–2.64		1.22	0.86–1.73
	No angry customers		1.00			1.00	
Autonomy	No		1.34 †	1.02–1.76		1.62 ‡	1.34–1.96
	Yes		1.00			1.00	
Ethical conflict	Not useful		1.67 ‡	1.27–2.21		1.56 ‡	1.29–1.87
	Useful		1.00			1.00	
Social relations and company conduct	No boss support		1.06	0.80–1.40		1.14	0.94–1.40
	Boss support		1.00			1.00	
	No colleague support **		1.14	0.85–1.51		1.58 ‡	1.30–1.93
	Colleague support		1.00			1.00	
	No HSI		1.21	0.94–1.57		1.40 ‡	1.18–1.66
	HSI		1.00			1.00	
	Unfair		1.75 ‡	1.35–2.28		1.44 ‡	1.20–1.72
	Fair		1.00			1.00	
6 Job instability	No new job prospects		1.11	0.87–1.43		1.19	0.99–1.43
	New job prospects		1.00			1.00	

* Odds ratios (OR) and 95% confidence intervals (95% CI) from the logistic regression analysis were adjusted for demographic variables (age, residential area, education level), basic working environment variables (employment status, working hours, wages), and the nine “key factor” sub-variables listed in the table. ^1^ Million Korean won (KRW); 1228 KRW = 1 US dollar (as of June 2020). † *p* < 0.05, ‡ *p* < 0.01. ** Effect on risk of depression differed significantly by gender.

**Table 3:** The interactive effects * of emotional demands and whether EDR and HSI exist on the risk of depression in males and females.

		Males (*n* = 2460)				Females (*n* = 5173)			
		EDR		HSI		EDR		HSI	
		Present	Absent	Not Provided	Provided	Present	Absent	Not Provided	Provided
Emotional Demands	Hide	1.1	0.88	1.21	0.99	1.80 ‡	1.24 †	1.90 ‡	1.46 ‡
	Not hide	0.73	1	1.2	1	0.99	1	1.51 ‡	1
	Angry	1.14	1.94 †	1.93	1.74	1.73 †	1.13	1.66 †	1.26
	Not angry	1.05	1	1.22	1	1.24 †	1	1.41 ‡	1

* Data are provided as odds ratios adjusted for demographic variables (age, residential area, education level), basic working environment variables (employment status, working hours, wages), and the eight “key factor” sub-variables other than the two comprising the interaction analysis. † *p* < 0.05, ‡ *p* < 0.01.

## Supplementary Materials

The following are available online at https://www.mdpi.com/1660-4601/17/14/5208/s1, Table S1: Six key factors associated with the risk of depression.
