# Supplementary material for: Effects of Emotional Labor Factors and Working Environment on the Risk of Depression in Pink-Collar Workers
Source: Int J Environ Res Public Health. 2020 Jul 19;17(14):5208. doi: 10.3390/ijerph17145208 (PMC7400525; doi:10.3390/ijerph17145208)
Supplement: Supplementary file 1 [file ijerph-17-05208-s001.pdf]

Supplementary Materials:

Table S1. Six key factors associated with the risk of depression.

		Males (*n* = 2460)				Females (*n* = 5173)			
		b[1]	Exp (b) [2]	95% CI [3]	*p* [4]	B [1]	Exp (b) [2]	95% CI [3]	*p* [4]
**Demographics**									
Age (y)	30-39	.5	1.66	1.13-2.44	.01	.5	1.66	1.19-2.31	<.01
	40-49	.45	1.57	1.05-2.36	.03	.6	1.82	1.35-2.46	<.01
	50-59	.29	1.33	0.85-2.07	.21	.85	2.33	1.73-3.14	<.01
	60+	.76	2.13	1.16-3.90	.01	.84	2.32	1.58-3.42	<.01
	15-29	.00	1.00			.00	1.00		
City size	Small	.18	1.19	0.94-1.51	.15	-.04	0.96	0.81-1.13	.61
	Big	.00	1.00			.00	1.00		
Education level	High school	.07	1.08	0.51-2.28	.84	.25	1.28	0.88-1.86	.19
	College or higher	.18	1.2	0.90-1.60	.22	-.02	0.98	0.80-1.20	.85
	Middle school or lower	.00	1.00			.00	1.00		
**Working Conditions**									
Work time (h)	43-50	.02	1.03	0.72-1.46	.89	-.08	0.93	0.74-1.16	.51
	>50	.29	1.33	1.00-1.78	.05	.02	1.02	0.83-1.25	.87
	<43	.00	1.00			.00	1.00		
Wages[5]	1.5-2.5	.58	1.78	1.01-3.12	.04	.11	1.12	0.90-1.40	.31
	2.5-4.0	.74	2.1	1.18-3.72	.01	0	1	0.73-1.35	.98
	>4.0	.78	2.19	1.19-4.05	.01	.23	1.26	0.88-1.81	.21
	<1.5	.00	1.00			.00	1.00		
Employment status	Temporary	.4	1.49	0.99-2.23	.05	.28	1.33	1.09-1.62	<.01
	Regular	.00	1.00			.00	1.00		
**Six key factors**	**Variables**								
High work demands	EDR	.94	1.01	0.76-1.34	.94	.02	1.26	1.03-1.53	.02
	No EDR	.00	1.00			.00	1.00		
Emotional demands	Hide Emotions*	-.003	1	0.78-1.28	.98	.31	1.36	1.15-1.62	<.01
	Not Hide Emotions	.00	1.00			.00	1.00		
	Angry Customers	.51	1.66	1.05-2.64	.03	.2	1.22	0.86-1.73	.26
	No Angry Customers	.00	1.00			.00	1.00		
Autonomy	No	.29	1.34	1.02-1.76	.03	.48	1.62	1.34-1.96	<.01

	Yes	.00	1.00			.00	1.00		
Ethical conflict	Not useful	.51	1.67	1.27-2.21	<.01	.44	1.56	1.29-1.87	<.01
	Useful	.00	1.00			.00	1.00		
Social relations and company conduct	No boss support	.06	1.06	0.80-1.40	.69	.13	1.14	0.94-1.40	.18
	Boss support	.00	1.00			.00	1.00		
	No colleague support*	.13	1.14	0.85-1.51	.39	.46	1.58	1.30-1.93	<.01
	Colleague support	.00	1.00			.00	1.00		
	No HSI	.19	1.21	0.94-1.57	.13	.34	1.4	1.18-1.66	<.01
	HSI	.00	1.00			.00	1.00		
	Unfair	.56	1.75	1.35-2.28	<.01	.36	1.44	1.20-1.72	<.01
	Fair	.00	1.00			.00	1.00		
Job insecurity	No new job prospects	.11	1.11	0.87-1.43	.4	.17	1.19	0.99-1.43	.07
	New job prospects	.00	1.00			.00	1.00		

[1] Beta coefficient. [2] Odds ratios from the logistic regression analysis adjusted for demographics (age, residential area, education level), basic working environment (employment status, working hours, wages), and the nine "key factor" sub-variables listed in the table. [3] Confidence interval. [4] All *p*- represents *p*-values which show the significance level of 6 key factors affecting the occurrence of risk of depression. [5] Millions of Korean won (KRW); 1,228 won = 1 US dollar (as of June 2020). * Significantly differed by gender.
